# Predictive Factors in OCT Analysis for Visual Outcome in Exudative AMD

**DOI:** 10.1155/2012/851648

**Published:** 2012-03-19

**Authors:** Maria-Andreea Gamulescu, Georgios Panagakis, Carmen Theek, Horst Helbig

**Affiliations:** ^1^University Eye Clinic, Franz-Josef-Strauss Allee 11, 93051 Regensburg, Germany; ^2^IFE Europe GmbH, Zeche Katharina 6, 45307 Essen, Germany

## Abstract

*Background*. Reliable predictive factors for therapy outcome may enable treating physicians to counsel their patients more efficiently concerning probability of improvement or time point of discontinuation of a certain therapy. *Methods*. This is a retrospective analysis of 87 patients with exudative age-related macular degeneration who received three monthly intravitreal ranibizumab injections. Visual acuity before initiation of intravitreal therapy and 4–6 weeks after last intravitreal injection was compared and related to the preoperative visualisation of continuity of the outer retinal layers as assessed by OCT: external limiting membrane (ELM), inner photoreceptor segments (IPS), junction between inner and outer segments (IS/OS), and outer photoreceptor segments (OPS). *Results*. Visual acuity increased in 40 of 87 (46.0%) patients, it remained stable in 25 (28.7%), and 22 (25.3%) patients had decreased visual acuity four to six weeks after triple intravitreal ranibizumab injections. No statistically significant predictive value could be demonstrated for grade of continuity of outer retinal layers concerning visual acuity development. *Conclusions*. In our series of AMD patients, grade of continuity of outer retinal layers was not a significant predictive value for visual acuity development after triple ranibizumab injections.

## 1. Introduction

Therapy of exudative age-related macular degeneration (AMD) with anti-vascular endothelial growth factor (VEGF) substances has evolved to an effective and widespread treatment in the last years, allowing preservation or even improvement of visual acuity in the short and long term [1–3]. Nevertheless, some patients fail to show improvement in visual acuity despite proven activity of the neovascular complex before beginning of therapy and anatomical success thereafter. Predictions concerning probability and amount of visual improvement still remain difficult, and counseling of patients is therefore challenging.

Improvements in optical coherence tomography (OCT) devices allow higher resolution of the imaged retina, and the assessment of the integrity of retinal layers becomes more and more feasible [4, 5]. Conclusions concerning the functionality of the single layers or their interaction may become possible and thus serve as a surrogative marker for the efficacy of certain therapies. Some publications already describe the importance of intact outer retinal layers, especially of the junction between inner and outer photoreceptor layers, for good visual acuity [6–10]. 

In the study presented, we investigated the predictive value of the outer retinal layers as visualized by OCT in relation to postoperative visual acuity in patients with exudative AMD receiving three intravitreal ranibizumab injections.

## 2. Methods

This is a retrospective chart review of 100 consecutive patients with treatment naïve subfoveal exudative AMD, who received three monthly intravitreal injections with ranibizumab (Lucentis, Novartis International AG, Basel, Switzerland). All patients were diagnosed with active subfoveal choroidal neovascular membrane (CNV) before treatment initiation. Patients received complete ophthalmologic exam including decimal best corrected visual acuity (BCVA), dilated fundus ophthalmoscopy, fundus photography, fluorescein angiography to confirm the diagnosis (Heidelberg Retina Angiograph 2, Heidelberg Engineering, Heidelberg, Germany), and OCT imaging (Spectralis, Heidelberg Engineering, Heidelberg, Germany). Decimal BCVA was converted to logMAR for statistical reasons. Four to six weeks after third intravitreal injection, patients routinely presented for follow-up examination including BCVA and OCT imaging. 

**Figure 1:**
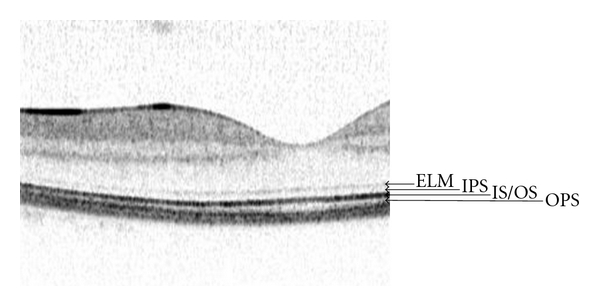
Spectral domain OCT scan of the retinal layers in a normal macula: ELM: external limiting membrane, IPS: inner photoreceptor segments, IS/OS: junction between inner and outer photoreceptor segments, and OPS: outer photoreceptor segments.

Preoperative OCT images were retrospectively evaluated concerning grade of continuity of the outer retinal layers, namely, the external limiting membrane (ELM), the inner photoreceptor segments (IPS), the junction between inner and outer photoreceptor segments (IS/OS), and the outer photoreceptor segments (OPS) (Figure 1). Gray-scale single-line (6 mm) horizontal OCT images centered on the fovea if visible, or approximating it, were used for this purpose, each image being averaged from 9 scans. Continuity of the outer retinal layers was evaluated over the whole width of the neovascular lesion. For ELM and IS/OS, continuity was classified in three categories: good visualization with continuity over the whole neovascular complex (+), moderate visualization with focal discontinuity (±), and low or no visualization over more than 50% of the neovascular complex (−). For IPS, and OPS, continuity was classified in good/smooth appearance (+) or low/ragged appearance (−), because no patient showed complete invisibility of IPS or OPS. 

Patients were divided into three groups depending on postoperative development of BCVA: group 1 had improvement of vision ≥1 logMAR line, group 2 stabilisation, and group 3 loss of visual acuity ≥1 logMAR line. A second analysis was carried out with patients in group 1 improving ≥3 lines, patients in group 2 stabilizing at ±2 lines, and patients in group 3 loosing ≥3 lines. Preoperative grade of continuity of outer retinal layers was then correlated to postoperative development of visual acuity.

Statistical analysis was performed using Fisher's exact test two sided with an *α*-level of 5% for the comparison of rates between groups.

Because of the retrospective and noninterventional study design, and because the data presented here cannot be related to personal patient data, the institutional ethics committee review board rated this study not to require an official approval.

## 3. Results

### 3.1. Overall Results

Charts and images of 100 consecutive patients were reviewed for this investigation. Of these, five patients missed the followup, and quality of OCT images of eight patients was too low for evaluation. Data of the remaining 87 patients was included in the final analysis. 59 female and 28 male patients, 42 right and 45 left eyes, were treated. All patients had naïve subfoveal exudative AMD in the studied eye that was categorized to the different CNV-subtypes according to fluorescein angiography: 10 of 87 patients (11.5%) had predominantly classic CNV, 18 (20.7%) had minimal classic CNV, and 59 (67.8%) had occult CNV. As graded by OCT imaging, 31 patients (35.6%) had intraretinal fluid accumulation with cystoid spaces only, 34 patients (39.1%) had subretinal fluid accumulation only, and 22 patients (25.3%) had combined intra- and subretinal fluid accumulation. 

Out of 87 patients, 40 (46.0%) experienced improvement in visual acuity ≥ 1 line, 25 (28.7%) remained stable, and 22 (25.3%) had decreased visual acuity ≥1 line four to six weeks after triple intravitreal ranibizumab injections. When considering improvement or loss of visual acuity of three or more lines, 18 (20.7%) patients improved, 64 (73.6%) remained stable, and 5 (5.7%) suffered from deterioration of visual acuity.

Overall (all patients taken together without discrimination concerning visual acuity), 27 (31%) out of 87 patients showed (+) ELM continuity, 30 (34.5%) patients (±) and 30 (34.5%) patients (−) ELM continuity. The IS/OS border was graded as showing (+) continuity in 15 (17.2%) patients, (±) in 33 (38.0%) patients, and (−) in 39 (44.8%) patients. Inner photoreceptors were (+) in 31 (35.6%) and (−) in 56 (64.4%) patients. Outer photoreceptors, respectively, were (+) in 9 (10.3%) and (−) in 78 (89.7%) patients. 

**Figure 2:**
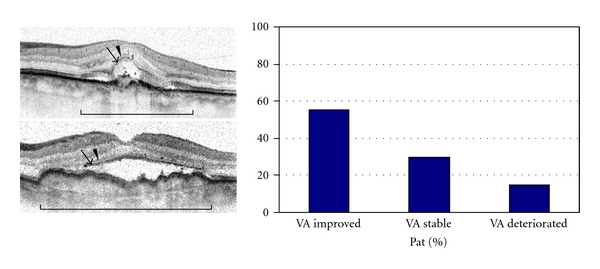
Group of patients with good continuity of ELM and IS/OS over the CNV lesion: exemplary baseline OCT images and final distribution of patients with improved, stable, or decreased vision after triple intravitreal ranibizumab therapy, (Brackets define the area of the CNV lesion. Arrowheads = ELM. Arrows = IS/OS border.).

Out of 27 patients with (+) ELM and considering visual gain or loss of one or more lines, 15 (55.6%) had improved visual acuity, 8 (29.6%) remained stable, and 4 (14.8%) lost vision (Figure 2). No patient had concurrent (−) IS/OS. 

**Figure 3:**
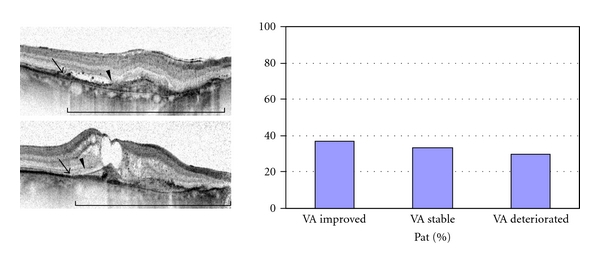
Group of patients with poor continuity of ELM and IS/OS over the CNV lesion: exemplary baseline OCT images and final distribution of patients with improved, stable, or decreased vision after triple intravitreal ranibizumab therapy (Brackets define the area of the CNV lesion. Arrowheads = ELM. Arrows = IS/OS border.).

Out of 30 patients with (−) ELM, 27 (90%) had also (−) IS/OS. Out of these 27 patients with (−) ELM and (−) IS/OS, 10 (37.1%) gained visual acuity, 9 (33.3%) remained stable, and 8 (29.6%) lost vision (Figure 3).

### 3.2. Predictive Value

#### 3.2.1. Good-Poor-No Continuity of Outer Retinal Layers

Concerning the predictive value of good versus poor versus no continuity of the outer retinal layers ELM, IS/OS, IPS, and OPS, statistical analysis did not reveal any significant value for any group when patients were stratified after visual acuity development (improved ≥ 1 line, stable, deteriorated ≥ 1 line): ELM *P* = 0.5879, IS/OS *P* = 0.7737, IPS *P* = 0.9579, and OPS *P* = 0.4951. Stratification of patients with improvement/loss of ≥3 lines did not result in significant predictive values, either, concerning ELM *P* = 0.2625, IS/OS *P* = 0.6987, and OPS *P* = 0.8147. Only the visibility of IPS was correlated with visual acuity development, when considering improvement/loss of ≥3 lines: *P* = 0.0169 (Table 1).

#### 3.2.2. Good Poor/No Continuity of Outer Retinal Layers

In a second assessment, the groups with (±) and (−) continuity of ELM and IS/OS, respectively, were combined and compared to patients with (+) continuity of ELM and IS/OS for the predictive value in relation to visual acuity development. 

Again, no significant value was obtained: ELM *P* = 0.3318, IS/OS *P* = 0.5457 for stratification ≥ 1 line and ELM *P* = 0.1987, IS/OS *P* = 0.5130 for stratification ≥ 3 lines. Likewise, no significant predictive value was found when visual acuity groups of stable and deteriorated visual acuity were combined and compared to the patients with improved visual acuity: ELM *P* = 0.2532, IS/OS *P* = 0.5789 for stratification ≥ 1 line and ELM *P* = 0.5679, IS/OS *P* = 0.7267 for stratification ≥ 3 lines.

#### 3.2.3. Good/Poor-No Continuity of Outer Retinal Layers

In a third assessment, the groups with (+) and (±) continuity of ELM and IS/OS, respectively, were combined and compared to patients with (−) continuity of ELM and IS/OS in the same manner. 

Again, no significant value was obtained: ELM *P* = 0.9178, IS/OS *P* = 0.7564 for stratification ≥ 1 line and ELM *P* = 0.2430, IS/OS *P* = 0.6383 for stratification ≥3 lines. 

Nor was any significance seen when visual acuity groups were combined: ELM *P* = 0.8221, IS/OS *P* = 0.5169 for stratification ≥ 1 line and ELM *P* = 0.1641, IS/OS *P* = 0.4255 for stratification ≥ 3 lines.

### 3.3. Influence of Fluid Accumulations

#### 3.3.1. On Visual Acuity Development

**Figure 4:**
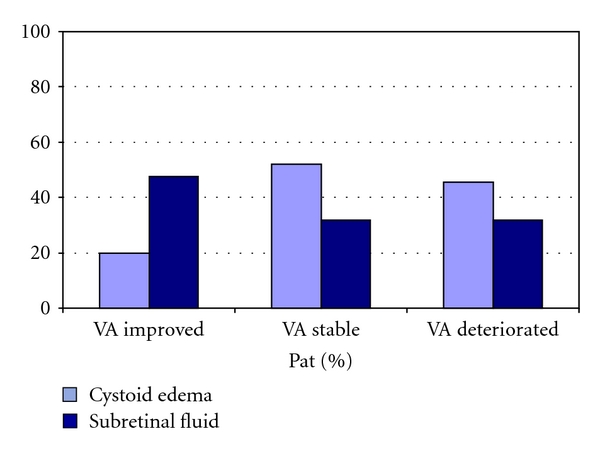
Distribution of patients with intraretinal cystoid edema versus subretinal fluid accumulation in OCT imaging on baseline, in relation to the final visual acuity.

Patients improving in vision 4–6 weeks after triple intravitreal ranibizumab injections had prorata substantially less intraretinal fluid accumulation and more subretinal fluid accumulation on baseline OCT: cystoid macular edema was seen in 20% of patients with improved vision compared to 52% and 45.5% of patients with stable or decreased vision, respectively. Subretinal fluid was present in 47.5% of patients with improved visual acuity, compared to 32% and 31.8% of patients with stable or decreased vision, respectively (Figure 4).

Combined intra- and subretinal fluid accumulations were present in 32.5% of patients with improved vision, and in 16% and 22.7% of patients with stable or decreased vision, respectively.

#### 3.3.2. On Continuity of Outer Retinal Layers

Presence of intraretinal fluid accumulations (cystoid edema) on baseline OCT was associated with less patients displaying good continuity of ELM and IS/OS on OCT images regardless of visual acuity development. In patients with improved vision and intraretinal cystoid spaces, ELM and IS/OS each was rated as displaying good continuity in only 7.5% of patients. In patients with subretinal fluid only, good continuity of ELM and IS/OS was seen in 30% and 12,5% of patients, respectively. This distribution was similar in the groups of patients with stable vision as well as patients with decreased vision (data not shown).

**Figure 5:**
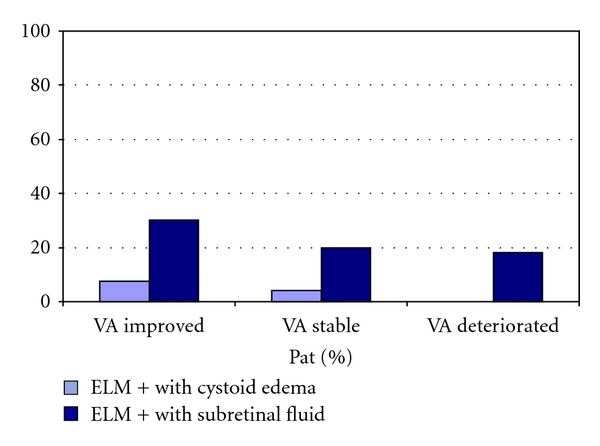
Percentage of patients displaying good continuity of external limiting membrane on baseline OCT and with either concurrent intraretinal cystoid edema or subretinal fluid accumulation, in relation to the final visual acuity.

Concerning only continuity of ELM and comparing groups of patients with improved versus stable versus decreased vision, good continuity of ELM despite intraretinal cystoid spaces was seen in 7.5%, 8%, and 0% of patients, respectively, while good continuity of ELM in the presence of subretinal fluid was seen in 30%, 20%, and 18.2%, respectively (Figure 5).

## 4. Discussion

Identification of predictive factors is of increasing importance as more efficacious therapies in a variety of ophthalmologic subfields become available. During the last years, predictive factors were primarily assessed as baseline morphologic features in the treated tissues or baseline patient characteristics such as age, BCVA, or previous interventions [11–13]. In AMD, for example, low initial BCVA, increased central retinal thickness in OCT and treatment naïve patients were found to be associated with an improvement in visual acuity after intravitreal bevacizumab therapy [12]. 

Due to advances in the resolution of OCT devices, in vivo imaging of retinal layers and, furthermore, assessment of integrity of those layers become more and more feasible in everyday routine [4, 5], and even subtle changes may be observed in a longitudinal fashion. In the outer retinal layers, the third hyperreflective line corresponding to the IS/OS junction represents the border between the highly organized outer photoreceptor segments and their inner myoid parts. The fine line overlying the IS/OS junction depicts the ELM and, thus, identifies the border between inner photoreceptor segments and outer nuclear layer comprising the photoreceptor cell bodies and—in the fovea—the apexes of the Müller cells [14]. 

In patients with cystoid macular edema associated with retinitis pigmentosa, Oishi and coworkers could show that the integrity of the IS/OS junction was strongly correlated with visual acuity, while total retinal thickness or thickness of the photoreceptor layer was not [6]. Also, visual acuity after therapy for different diseases was found to correlate to the postoperative integrity of the IS/OS junction [7–10]. This finding is not surprising as the integrity of IS/OS junction and visualization of this line in OCT imaging indicates integrity of the photoreceptor system. Furthermore, integrity of ELM would suggest integrity of both inner photoreceptor segments and outer nuclear layer, the thickness of which was also correlated with good visual acuity [8]. 

However, only few publications are available up to now assessing the predictive value of the continuity of outer retinal layers. Suh and coworkers showed that eyes with disruption of IS/OS junction on preoperative OCT had significantly lower postoperative BCVA after removal of epiretinal membranes [15]. However, although visual acuity may be biased as some patients also had cataract operation and/or triamcinolone injection at the end of the operative procedure, this study hints to the importance of integrity of IS/OS junction for good visual performance. In addition, Wakabayashi and co-workers correlated the intact ELM and IS/OS junction one month after successful retinal detachment repair with further visual acuity improvement. Furthermore, they stress the importance of an intact ELM both for final visual acuity development and also for possible restoration of disrupted IS/OS junction in the followup. They postulate that restoration of photoreceptors is possible as long as the degenerative changes have not yet reached the cell bodies [16]. In a recent publication, Wolf-Schnurrbusch and colleagues showed that visual acuity improvement in patients with central retinal vein occlusion 4 weeks after single intravitreal anti-VEGF injection is associated with an intact ELM [17].

In our study population of exudative AMD patients, the grade of continuity, and thereby integrity, of none of the outer retinal layers including ELM and IS/OS junction was associated with visual acuity development when considering change of visual acuity of more than one line. The single significant value concerning the IPS when considering change in visual acuity of more than three lines might have been biased by the very small number of patients in these groups and will have to be reevaluated in a larger series. On the other hand, although initial OCT imaging showed ELM and IS/OS discontinuity in 34.5% and 44.8% of patients, respectively, only 25.3% had deterioration in postoperative visual acuity. Likewise, while a significant part of patients (89.7%) showed poor/ragged appearance of OPS on initial OCT images, visual acuity nevertheless improved in 46% after ranibizumab injections. This may support the hypothesis that disrupted or ragged outer photoreceptor layers may regenerate with time and thereby allow patients to improve in visual acuity [16]. Also, in a study population comprising exudative AMD, disorganisation of retinal layers is much more extensive than in the above-mentioned publications of epiretinal membrane peeling or retinal detachment surgery [15, 16]. In our series of exudative AMD patients, intraretinal cystoid edema was associated with a poorer visual prognosis as well as with lower grades of continuity of outer retinal layers than subretinal fluid accumulation. On the other hand, an increased amount of intraretinal fluid accumulation before beginning of therapy may reduce the visibility and assessibility of the retinal layers by increased absorption and backscattering of signals, thereby biasing the predictive potency of the images: while good visualization and continuity of ELM and IS/OS junction is associated with a higher probability of improved vision in our study, although not statistically significant, poor visualization and continuity does not allow any prediction concerning visual acuity development. Therefore, presence of intraretinal cystoid spaces should caution in predictions of visual acuity.

The limitations of this study are its retrospective nature and highly mixed patient population of everyday routine out-patient service, as well as the short followup of only 4 to 6 weeks after upload injections and the averaging of only 9 scans per OCT image. Nevertheless, as anti-VEGF-therapy shows a steep improvement in vision during upload-injections and a plateau forming afterwards, we felt that even this short followup would give good information about the predictive value of OCT images.

Therefore, and although most publications dealing with the predictive value of outer retinal layers stress the importance of integrity of outer retinal layers for good visual acuity, we would like to caution for the reverse, as not all patients with poor visualization or even discontinuity of outer retinal layers deteriorate in the followup. More detailed and specific factors are therefore needed for exact prediction of postoperative visual development.

## Figures and Tables

**Table 1:** 

		VA improved ±1 line (±*3 lines*)	VA stable ±1 line (±*3 lines*)	VA deteriorated ±1 line (±*3 lines*)
	+			
ELM	±	*P* = 0.5879 (*P* = 0.2625)		
	−			

	+			
OS/OS	±	*P* = 0.7737 (*P* = 0.6987)		
	−			

IPS	+	*P* = 0.9579 (*P* = 0.0169)		
	−			

OPS	+	*P* = 0.4951 (*P* = 0.8147)		
	−			
